# Assessment of liver fibrosis and associated risk factors in HIV-infected individuals using transient elastography and serum biomarkers

**DOI:** 10.1186/1471-230X-12-27

**Published:** 2012-03-27

**Authors:** Johannes Vermehren, Annika Vermehren, Axel Mueller, Amina Carlebach, Thomas Lutz, Peter Gute, Gaby Knecht, Christoph Sarrazin, Mireen Friedrich-Rust, Nicole Forestier, Thierry Poynard, Stefan Zeuzem, Eva Herrmann, Wolf Peter Hofmann

**Affiliations:** 1 Medizinische Klinik 1, Klinikum der J. W. Goethe-Universität, Frankfurt am Main, Germany; 2 Infektiologikum, Frankfurt am Main, Germany; 3 Hôpital Pitié Salpétrière, Paris, France; 4 Institut für Biostatistik und mathematische Modellierung, Fachbereich Medizin der J. W. Goethe-Universität, Frankfurt am Main, Germany; 5 POLIKUM Gesundheitszentren, Berlin, Germany

**Keywords:** HIV, HCV, co-infection, cART, Hepatotoxicity, Transient elastography, Fibrotest, Liver enzymes

## Abstract

**Background:**

Liver fibrosis in human immunodeficiency virus (HIV)-infected individuals is mostly attributable to co-infection with hepatitis B or C. The impact of other risk factors, including prolonged exposure to combined antiretroviral therapy (cART) is poorly understood. Our aim was to determine the prevalence of liver fibrosis and associated risk factors in HIV-infected individuals based on non-invasive fibrosis assessment using transient elastography (TE) and serum biomarkers (Fibrotest [FT]).

**Methods:**

In 202 consecutive HIV-infected individuals (159 men; mean age 47 ± 9 years; 35 with hepatitis-C-virus [HCV] co-infection), TE and FT were performed. Repeat TE examinations were conducted 1 and 2 years after study inclusion.

**Results:**

Significant liver fibrosis was present in 16% and 29% of patients, respectively, when assessed by TE (≥ 7.1 kPa) and FT (> 0.48). A combination of TE and FT predicted significant fibrosis in 8% of all patients (31% in HIV/HCV co-infected and 3% in HIV mono-infected individuals). Chronic ALT, AST and γ-GT elevation was present in 29%, 20% and 51% of all cART-exposed patients and in 19%, 8% and 45.5% of HIV mono-infected individuals. Overall, factors independently associated with significant fibrosis as assessed by TE (OR, 95% CI) were co-infection with HCV (7.29, 1.95-27.34), chronic AST (6.58, 1.30-33.25) and γ-GT (5.17, 1.56-17.08) elevation and time on dideoxynucleoside therapy (1.01, 1.00-1.02). In 68 HIV mono-infected individuals who had repeat TE examinations, TE values did not differ significantly during a median follow-up time of 24 months (median intra-patient changes at last TE examination relative to baseline: -0.2 kPa, p = 0.20).

**Conclusions:**

Chronic elevation of liver enzymes was observed in up to 45.5% of HIV mono-infected patients on cART. However, only a small subset had significant fibrosis as predicted by TE and FT. There was no evidence for fibrosis progression during follow-up TE examinations.

## Background

Effective and long-term combination antiretroviral therapy (cART) has substantially decreased morbidity and mortality in human immunodeficiency virus (HIV) infected individuals [1,2]. The incidence of acquired immune deficiency syndrome (AIDS)-defining events has continuously declined over the past years and liver diseases, mainly due to co-infection with the hepatitis C virus (HCV) and/or hepatitis B virus (HBV) or presence of metabolic syndrome-associated non-alcoholic steatohepatitis have emerged as key issues within the HIV infected population [2]. In addition, cART-related hepatotoxicity may contribute to hepatic injury in both HIV mono-infected individuals and those with hepatitis co-infection. Albeit not clearly defined, cART-related hepatotoxicity may occur in the context of acute events such as hypersensitive reactions and lactic acidosis or may be associated with progressive liver damage resulting in non-alcoholic steatohepatitis and significant fibrosis [3,4]. In a recent study, chronic alanine aminotransferase (ALT) elevations have been observed in 16% of HIV mono-infected individuals during cART but their clinical significance over time is poorly understood [5].

The effects of long-term cART on liver fibrosis in patients with or without hepatitis co-infection and the involvement of the metabolic syndrome continue to be controversially discussed. While on the one hand, long-term cART was found to be a protective factor for liver fibrosis in some studies [6,7], other findings suggest that exposure to dideoxynucleosides and other nucleoside analog reverse-transcriptase inhibitors (NRTIs) is associated both with chronic elevation of aspartate aminotransferase (AST) and/or ALT levels and liver fibrosis [5,8].

Liver biopsy remains the gold standard for the accurate assessment of liver fibrosis. However, the procedure is invasive and may be associated with severe adverse events [9]. In addition, sampling errors as well as observer variability may limit the reliability of the procedure. Therefore, blood tests (e.g., AST-to-platelet ratio index [APRI] and Fibrotest [FT]) have been developed to non-invasively predict the extent of liver fibrosis in a variety of liver diseases [10,11].

Likewise, transient elastography (TE), an ultrasound-based method, which estimates liver fibrosis by measuring liver stiffness has become widely available as a quick and reliable non-invasive means to assess liver fibrosis [12]. Several studies have shown that there is a good correlation for the prediction of advanced liver fibrosis and cirrhosis between blood tests, TE and liver histology [13-17].

In this study, we sought to non-invasively assess the prevalence of liver fibrosis and associated risk factors in a cohort of HIV-infected individuals as assessed by TE alone, FT alone or TE and FT combined.

## Methods

### Patient population

All patients aged 18 and over with chronic HIV infection who attended the hepatology outpatient clinic at the J. W. Goethe University Hospital between December 2008 and June 2009 were offered non-invasive assessment of liver fibrosis using TE and blood tests (cross-sectional study). In addition, all individuals were offered repeat TE measurements at one year (in 2010) and two years (in 2011) after study inclusion (longitudinal study).

Demographic data, including age, gender, ethnicity, and BMI (defined as weight in kilograms divided by the height in meters squared) were recorded for each individual patient at baseline in the cross-sectional study. In addition, most recent virologic data, including hepatitis B surface antigen (HBsAg) status, HCV antibody status plus HCV RNA, HIV RNA, and CD4+ T-cell count as well as detailed history of prior antiretroviral therapy was also recorded. Patients with known or suspected liver disease other than HCV and/or HBV and with past or present alcohol abuse (more than two standard drinks [13.7 g] for men or 1 standard drink for women per day) were excluded from the study.

The study was performed in accordance with the Declaration of Helsinki and was approved by the local ethics committee. All patients had signed a written informed consent prior to study inclusion.

### Blood tests

Blood tests were performed in all patients after an overnight fast of at least 12 h on the same day that TE was performed. The following blood parameters were taken: AST, ALT, γ-glutamyl-transferase (γGT), alkaline phosphatase, total bilirubin, platelet count, α2-macroglobulin, apolipoprotein A1, haptoglobin, glucose, insulin, total cholesterol, triglycerides, and haptoglobin. Enzymatic activity was measured at 37°C in accordance with the International Federation of Clinical Chemistry Standards. Serum samples for insulin determination were immediately refrigerated at 4°C and shipped for testing on the same day.

Chronic elevation of liver enzymes was defined as an ALT, AST or γ-GT level greater than the age-adjusted upper limit of normal at two or more consecutive semiannual visits, in line with previously recommended definitions [5].

The commercially available Fibrotest (FT; BioPredictive, Paris, France) was performed using a patented algorithm that includes α2-macroglobulin, apolipoprotein A1, haptoglobin, γ-GT and total bilirubin, adjusted for age and gender [10]. Security algorithms requiring the exclusion of patients at high risk for false-positive or false-negative results were respected. Cut-off values for the diagnosis of significant fibrosis (> 0.48) and cirrhosis (≥ 0.75) were chosen in line with the manufacturer's recommendations.

Finally, the homeostasis model assessment (HOMA) insulin resistance (IR) index (fasting plasma insulin [μU/mL] × fasting plasma glucose [mg/dL]/405) was also recorded in each patient.

### Transient elastography (TE)

All TE examinations were performed after an overnight fast of at least 12 h.

TE (FibroScan^®^, Echosens, Paris, France) involves a probe that includes an ultrasound transducer mounted on the axis of a vibrator. A vibration wave excited by the vibrator induces an elastic shear wave that propagates through the liver tissue. The velocity of these propagations is measured by ultrasound and directly correlates with liver stiffness. The results are expressed in kilopascals (kPa) [18].

TE measurements were considered reliable when at least 10 successful measurements with a success-rate of at least 60% and an interquartile range of 30% or lower were achieved, according to the manufacturer's instructions.

For statistical analyses, TE results were assigned to different stages of liver fibrosis according to the semiquantitative histological staging system of METAVIR. The predefined cut-off values were ≥ 7.1 kPa for significant fibrosis (corresponding to ≥ F2) and ≥ 12.5 kPa for cirrhosis (corresponding to ≥ F4) in line with previous recommendations [19-21].

### Combination of noninvasive methods

To improve the diagnostic accuracy of noninvasive methods, a combination of TE and FT was determined based on an algorithm recently proposed by Castera and co-workers (Bordeaux algorithm) [22]. Cut-off values for the combination of TE and FT were ≥ 7.1 kPa plus > 0.48 for the detection of significant fibrosis and ≥ 12.5 kPa plus ≥ 0.75 for the detection of cirrhosis, respectively.

### Statistical analysis

All statistical analyses were carried out using the SPSS Statistics Software Package for Windows, version 19.0 (SPSS, IBM, Somers, NY, USA) or GraphPad Prism for Windows, version 4.02 (GraphPad Software, La Jolla, CA, USA).

Clinical and laboratory characteristics of patients are given as mean ± standard deviation or median and range, as appropriate.

For categorical variables, the chi-square test or the Fisher's exact test was performed. For continuous variables, the Student's t-test or the Mann-Whitney U-test was used. Intra-group comparisons were made using the Wilcoxon's test for paired data. All tests were two-tailed and a p-value < 0.05 was judged to be statistically significant.

For association analyses, univariate and multivariate models were employed. Parameters with a p-value < 0.05 in the univariate analysis were included in a multivariate logistic regression model based on the backward stepwise Wald method.

## Results

### Baseline patient characteristics

In total, 202 individuals with HIV infection had liver fibrosis assessed by TE and blood tests. The respective examinations were considered valid in all patients.

The main patient characteristics are summarized in Table 1. The mean age was 47 ± 9 years. The proportion of men was 79% (n = 159), and 94% of the subjects were of Caucasian ethnicity (n = 190). The mean duration of HIV infection was 13 ± 7 years. The majority of patients were on antiretroviral therapy (89%, n = 179). The mean CD4+ T-cell count was 591 ± 254 cells/μl and plasma HIV RNA was below the limit of detection in 77% of patients (n = 156). HCV co-infection was present in 17% (n = 35; mean duration of infection, 13 ± 5 years) of patients and 9% (n = 18; mean duration of infection, 15 ± 5 years) were HBsAg positive.

**Table 1:** Main baseline characteristics of the study population (n = 202) by liver fibrosis stage using transient elastography (TE)

Variable	All patients	Patients with TE < 7.1 kPa	Patients with TE ≥ 7.1 kPa	p-value
Mean age, years	47 ± 9	46.7 ± 9	49 ± 9	0.20
Male gender, n (%)	159 (79)	135 (80)	24 (73)	0.36
Caucasian Ethnicity, n (%)	190 (94)	157 (93)	33 (100)	0.87
				
CD4 count (cells/μl), mean ± SD	591 ± 254	591 ± 240	589 ± 323	0.57
Estimated duration of HIV infection in years, mean ± SD	13 ± 7	13 ± 7	15 ± 6	0.70
HBV co-infection, n (%)	18 (9)	13 (8)	5 (15)	0.18
HCV co-infection, n (%)	35 (17)	16 (10)	19 (58)	**< 0.0001**
Chronic ALT elevation, n (%)	55 (27)	38 (23)	17 (52)	**0.001**
Chronic AST elevation, n (%)	38 (19)	19 (11)	19 (58)	**< 0.0001**
Chronic γ-GT elevation, n (%)	98 (49)	73 (43)	25 (76)	**0.001**
				
Exposure to cART, n (%)	179 (89)	148 (88)	31 (94)	0.38
months on NRTI				
mean ± SD	188 ± 135	186 ± 138	199 ± 124	0.60
months on NNRTI				
mean ± SD	36 ± 44	38 ± 47	32 ± 34	0.68
months on PI				
mean ± SD	90 ± 114	83 ± 108	131 ± 135	0.72
Months on d-drugs				
mean ± SD	32 ± 48	27 ± 44	55 ± 64	**0.006**
				
BMI (kg/m^2^), mean ± SD	23.4 ± 3.0	23.2 ± 2.8	24.7 ± 3.7	0.06
HOMA-IR, mean ± SD	2.4 ± 2.3	2.1 ± 1.8	3.8 ± 4.0	**0.006**
Total serum cholesterol (mg/dl),				
mean ± SD	203 ± 45	209 ± 45	176 ± 34	**< 0.0001**

P-values are given for differences between patients with TE values < 7.1 kPa (n = 169) vs. ≥ 7.1 kPa (n = 33)

cART, combination antiretroviral therapy; NRTI nucleoside reverse transcriptase inhibitors; NNRTI, non-nucleoside reverse transcriptase inhibitors; PI, protease inhibitors; d-drugs, stavudine, didanosine, or zalcitabine

Patients with HIV/HCV co-infection were more often former iv-drug users (47% vs. 1%, p < 0.0001; data not shown) and were longer infected with HIV than those with HIV mono-infection (17 vs. 13 years, p = 0.002). In addition, HIV/HCV co-infected patients were longer exposed to dideoxynucleosides (stavudine, didanosine, or zalcitabine), compared to patients with HIV mono-infection (49 vs. 28 months, p = 0.048).

### Liver fibrosis staging - cross-sectional study

The median liver stiffness in the study population was 4.9 kPa (range, 2.4-36.8; mean ± SD, 6.1 ± 4.5 kPa). In total, 33 (16%) and 10 (5%) patients had TE values corresponding to significant liver fibrosis (≥ 7.1 kPa) and cirrhosis (≥ 12.5 kPa), respectively.

The main baseline patient characteristics according to liver stiffness values ≥ 7.1 kPa vs. < 7.1 kPa are shown in Table 1. Patients with TE values ≥ 7.1 kPa corresponding to significant (or higher) liver fibrosis were more often co-infected with HCV than patients with TE values < 7.1 kPa (p < 0.0001). In addition, chronic elevation of ALT (p = 0.001), AST (p < 0.0001) and γ-GT (p = 0.001), high HOMA-IR (p = 0.006) and cumulative exposure to dideoxynucleoside therapy (p = 0.006) was more commonly observed in patients with presumed significant fibrosis as compared to those without significant fibrosis. In contrast, total serum cholesterol levels were lower in patients with significant fibrosis (p < 0.0001).

According to FT values, significant liver fibrosis (> 0.48) and cirrhosis (≥ 0.75) were present in 59 (29%) and 16 (8%) patients, respectively.

When both TE and FT were combined, 16 (8%) and 5 (3%) patients had predicted significant fibrosis (≥ 7.1 kPa + > 0.48) or cirrhosis (≥ 12.5 kPa + ≥ 0.75), respectively. Finally, after excluding viral hepatitis, only 5 (3%) and 1 (0.6%) remained to have significant fibrosis and cirrhosis according to the combined tests. On the contrary, 11 (31%) and 4 (11%) of HIV/HCV co-infected individuals had predicted significant fibrosis and cirrhosis according to the test combination.

### Chronic elevation of liver enzymes

Chronic elevation of ALT, AST and γ-GT levels was present in 27% (n = 55), 19% (n = 38) and 49% (n = 98) of all patients, respectively (Table 1). In patients on cART, 29% (n = 52), 20% (n = 36) and 51% (n = 92) had chronic elevated ALT, AST and γ-GT levels. After excluding viral hepatitis (HBV and HCV) in cART-exposed patients, 19% (n = 25), 8% (n = 11) and 45.5% (n = 60) still had elevated liver enzymes. Among patients with chronic γ-GT elevation, 45% (n = 41) were taking the non-nucleoside reverse transcriptase inhibitor nevirapine, a drug commonly associated with γ-GT elevation.

Baseline characteristics associated with significant liver fibrosis/cirrhosis as assessed by TE, FT or combination of the two.

Univariate analysis (OR [95% CI] showed that co-infection with HCV (12.98 [5.49-30.71]), chronic elevation of ALT (3.66 [1.69-7.93]), AST (10.71 [4.63-24.80]) and γ-GT (4.11 [1.75-9.64]) and cumulative exposure to certain antiretrovirals including protease inhibitors (1.00 [1.00-1.04]) and dideoxynucleosides (1.01 [1.00-1.02]) were all associated with significant liver fibrosis as measured by TE alone. Furthermore, metabolic factors such as high HOMA-IR (1.28 [1.09-1.49]), high BMI (1.17 [1.04-1.33]) and low serum cholesterol (0.98 [0.97-0.99]) were also associated with liver stiffness ≥ 7.1 kPa. Factors that remained independently associated with significant fibrosis in the multivariate analysis were co-infection with HCV, chronic elevation of AST and γ-GT, cumulative exposure to dideoxynucleosides and low serum cholesterol. Results of the multivariate analyses (OR; 95% CI) are displayed in Table 2.

**Table 2:** Factors associated with significant fibrosis according to TE (≥ 7.1 kPa), Fibrotest (> 0.48) and TE and Fibrotest combined

	TE F ≥ 2		FT F ≥ 2		TE + FT F ≥ 2	
**Variable**	**Multivariate analysis** **OR (95% CI)**	**p-value**	**Multivariate analysis** **OR (95% CI)**	**p-value**	**Multivariate analysis** **OR (95% CI)**	**p-value**

HCV-RNA positive	7.29 (1.95-27.34)	0.003				
Chronic AST elevation	6.58 (1.30-33.25)	0.023	2.72 (1.18-6.27)	0.019	137.04 (9.57-1961.89)	< 0.0001
Chronic GGT elevation	5.17 (1.56-17.08)	0.007	2.69 (1.30-5.57)	0.008	17.92 (1.52-210.98)	0.022
Total serum cholesterol	0.98 (0.96-0.99)	0.001			0.96 (0.93-0.99)	0.004
Cumulative d-drug exposure (in months)	1.01 (1.00-1.02)	0.035				
Saquinavir			1.02 (1.00-1.03)	0.016	1.03 (1.00-1.06)	0.046

Data of the multivariate analysis are shown

TE, transient elastography; FT, Fibrotest; F ≥ 2, significant liver fibrosis; d-drug exposure, exposure to dideoxynucleosides

Factors associated with significant fibrosis predicted by FT alone were age 1.04 [1.00-1.08]), HCV co-infection (3.77 [1.77-8.01]), chronic elevation of ALT (3.47 [1.76-6.70]), AST (4.72 [2.25-9.90]) and γ-GT (3.44 [1.80-6.57]) levels and cumulative exposure to certain antiretrovirals, including stavudine (1.013 [1.00-1.03]) lopinavir (1.012 [1.00-1.02]) saquinavir (1.022 [1.01-1.04]) protease inhibitors (1.004 [1.00-1.01]) were all associated with significant fibrosis predicted by FT. HOMA-IR (1.22 [1.06-1.41]) was the only metabolic factor associated with FT > 0.48. Factors remaining independently associated with significant fibrosis in the multivariate analysis (OR; 95% CI) were chronic AST and γ-GT elevation as well as time on saquinavir (Table 2).

Finally, univariate analysis showed that co-infection with HCV (14.85 [4.75-46.47]), chronic ALT [7.10 2.34-21.54]), AST (47.25 [10.12-220.92]), γ-GT (18.61 [2.41-143.84]) and cumulative exposure to antiretrovirals, including protease inhibitors (1.004 [1.00-1,01]) and dideoxynucleosides (1.01 [1.00-1.02]) were associated with combined prediction of significant fibrosis by TE and FT. Among metabolic factors, high BMI (1.24 [1.05-1.45]), high HOMA-IR (1.35 [1.13-1.61]) and low serum cholesterol (0.98 [0.96-0.99]) were also associated with significant fibrosis. In the multivariate analysis (OR; 95% CI), chronic AST and γ-GT elevation, low serum cholesterol and exposure to saquinavir remained independently associated with significant fibrosis (Table 2).

### Assessment of liver fibrosis at follow-up - longitudinal study

Sixty-eight patients with HIV mono-infection had at least one repeat TE examination (median time interval to first examination, 21 months; range, 9-27 months) and 20 of these 68 patients had two repeat TE examinations (median time interval to second examination, 24 months; range, 19-26 months). When pooling the last available TE examination for each of the 68 patients, the median follow-up time was 24 months (range, 9-27 months).

**Figure 1:**
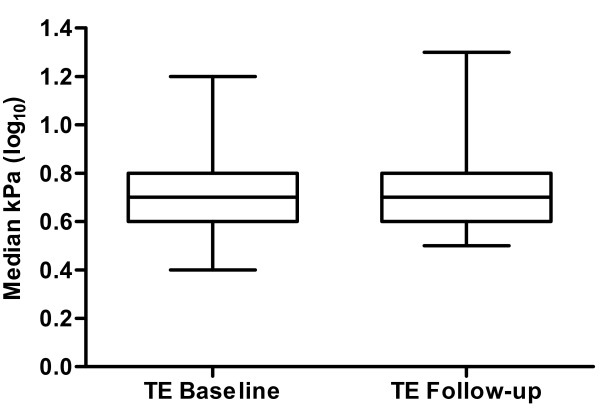
**Box plots of median liver stiffness (TE) values at baseline and follow-up in 68 patients with HIV mono-infection (p = 0.20)**. All data were log_10_-transformed. The top and bottom of each box represent the first and third quartiles respectively. The middle line represents the median. Pooled follow-up TE values are shown for the last available TE examination in each patient. Median follow-up time was 24 months (range, 9-27 months).

The median liver stiffness at the end of follow-up (last examination available in each patient) was 4.9 kPa (range, 2.9-20.2 kPa; Figure 1).

The 68 patients without viral hepatitis who had at least one repeat TE examination were not significantly different from those with only one TE determination at baseline for demographic characteristics, including age, gender, BMI, ethnicity and risk behavior (data not shown).

Among 63/68 patients who had liver stiffness values < 7.1 kPa at baseline, 55 (87%) still had TE values < 7.1 kPa at the end of follow-up, whereas 8 patients (13%) had increased liver stiffness. Furthermore, 55/57 patients (96.5%) with liver stiffness values < 7.1 kPa at follow-up also had values below this threshold at baseline. Despite these individual changes relative to the 7.1 kPa threshold, median intra-patient liver stiffness changes at the end of follow-up were not significantly different compared to baseline in the 68 patients (-0.2 kPa; p = 0.20).

## Discussion

The impact of cART and other risk factors on the development of liver fibrosis in patients infected with HIV has been subject to controversial debate over the past years. Several studies indicate that long-term cART may worsen liver fibrosis in patients with HIV/HCV co-infection and that this effect is mostly seen with NRTIs. However, effective control of HIV, especially through the introduction of PIs has recently been shown to be associated with slower liver fibrosis progression in patients with HIV/HCV co-infection who underwent paired liver biopsies [6]. Moreover, little is known about risk factors in patients without hepatitis co-infection and few data exist on liver-related long-term complications of cART [3].

In our prospective study of HIV infected individuals, mostly without hepatitis co-infection (76% of all patients), elevated ALT, AST and γ-GT levels were observed in 19%, 8% and 45.5% of those without viral hepatitis, suggestive of the presence of cART-related hepatotoxicity and/or metabolic disorders. However, only one patient fulfilled the Hy's rule criteria (ALT > 3xULN and total bilirubin > 2xULN) [23]. Despite there being no generally accepted definition of drug-induced hepatotoxicity, elevations of liver transaminases (ALT and AST) are currently considered as the best indicator [5,24].

When using TE for fibrosis assessment, 16% and 5% of patients were predicted to have significant liver fibrosis and cirrhosis, respectively. Interestingly, this was not exclusively attributable to HCV co-infection, as 42% and 30% of patients with liver stiffness suggestive of significant fibrosis and cirrhosis were indeed HCV RNA negative.

Recently, Castera and co-workers have proposed an algorithm for non-invasive fibrosis assessment that includes a combination of TE and FT (Bordeaux algorithm). When combining the two modalities, the presence or absence of significant liver fibrosis and cirrhosis was predicted with an accuracy of 87.7% and 95.7%, respectively [22].

When we applied the above-mentioned criteria to our data, only 8% and 3% of our patients had significant liver fibrosis or cirrhosis. Moreover, in patients with HIV mono-infection, the proportion of patients with significant fibrosis and cirrhosis was even smaller (3% and 0.6%, respectively).

Thus, despite a high prevalence of chronic elevated ALT, AST or γ-GT levels, there was only evidence for liver fibrosis in a small proportion of HIV mono-infected patients. It should be noted however, that the Bordeaux algorithm has not yet been validated in HIV-infected individuals with or without HCV co-infection.

In a longitudinal analysis, liver stiffness values did not differ significantly over a median follow-up time of 24 months in patients with HIV mono-infection, indicating that patients on cART may not be at risk of progressive liver disease. However, the rather short follow-up period may impose a latency bias on this observation as the actual mean rate of liver fibrosis progression within one year has been estimated to be as low as 0.085-0.120 for fibrosis stages derived according to the METAVIR scoring system in patients with chronic HCV infection [25] and it may be even lower in patients with drug-induced fibrosis or steatohepatitis.

Overall, in patients in whom liver fibrosis was predicted by TE, factors independently associated with significant fibrosis included HCV-coinfection, chronic AST and γ-GT elevation as well as exposure to dideoxynucleosides and these results are in line with previous observations [8,19]. With regard to cART-related hepatotoxicity, several specific antiretroviral agents such as nevirapine, zidovudine, stavudine, and other nucleoside analogs have been associated with liver fibrosis progression, especially in patients with HIV/HCV co-infection [8,26,27]. Nevirapine in particular has been associated with ALT and γ-GT elevation and this may account for the high prevalence of chronic γ-GT elevation in our study as almost half of those with γ-GT elevation were taking nevirapine at the time. This may also explain the higher percentage of significant liver fibrosis predicted by Fibrotest that combines the results of five blood serum tests, including γ-GT.

The potentially hepatotoxic effect of dideoxynucleoside-induced mitochondrial toxicity has been discussed previously [28,29]. The reduction of mitochondrial beta-oxidation of fatty acids may lead to hepatic steatosis, which, over time, can lead to chronic inflammation and liver fibrosis [30,31]. It must be noted, however, that dideoxynucleosides have been widely replaced by newer NRTIs with superior liver safety profiles and the use of dideoxynucleosides is rapidly diminishing. This may also explain why there was no increase in liver fibrosis observed in our longitudinal study. Moreover, when using both TE and FT, cumulative time on dideoxynucleosides was only associated with significant fibrosis in the univariate analysis.

While liver steatosis and steatohepatitis may develop as a direct consequence of NRTI-toxicity, these conditions can also be associated with cART-related metabolic disorders, including hyperglycemia, insulin resistance, dyslipidemia, and obesity. Indeed, high HOMA-IR and BMI were associated with predicted liver fibrosis when assessed by TE and/or FT. However, their impact did not remain significant in the multivariate models.

In several studies, excessive alcohol use was also shown to be associated with liver fibrosis in the HIV population [8,32]. In the present study, we excluded patients with alcohol abuse (more than 2 standard drinks in men/one standard drink in women) in order to minimize the alcohol-related impact on liver fibrosis. Indeed, alcohol consumption below this threshold was not associated with increased liver stiffness in this study.

A limitation of the present study is the lack of fibrosis assessment by liver biopsy. However, the role of liver biopsy as "gold-standard" is subject to increasing debate, owing to possible side effects as well as frequently occurring sampling errors and variability of sample interpretation [33]. Furthermore, liver biopsy is rarely recommended in patients with HIV mono-infection.

## Conclusions

Taken together, our results showed that significant fibrosis or cirrhosis was present in a small subset of patients with HIV mono-infection. However, no evidence was observed for fibrosis progression over time. Chronic AST and γ-GT elevations as well as long-term dideoxynucleoside therapy were independent risk factors for those with liver stiffness values ≥ 7.1 kPa and highlight the role of drug hepatotoxicity in fibrosis development. Longer follow-up studies may be necessary to show whether cART-associated elevation of liver enzymes leads to increased liver related morbidity and mortality.

## Competing interests

MFR has served as a clinical investigator and member of the speakers' bureau for Echosens. TP has a capital interest in Biopredictive, the company that markets Fibrotest. The patent for this biomarker is owned by a public organization (Assistence Publique Hôpitaux de Paris, France). All other authors have no conflicts to declare.

## Authors' contributions

JV performed TE examinations, participated in the design of the study, analysis of the data, performed the statistical analysis and drafted the manuscript. AV performed TE examinations and participated in the design and analysis of the study. AM, AC, TL, PG, & GK participated in the design and analysis of the data and helped with patient recruitment. MFR participated in the design and analysis of the data and helped to draft the manuscript. NF participated in the design and analysis of the data. TP participated in the design and analysis of the data. SZ participated in the design and analysis of the data. EH participated in the study design and performed the statistical analysis. WPH was responsible for the entire study design, performed the statistical analysis and helped to draft the manuscript. All authors read and approved the final manuscript.

## Pre-publication history

The pre-publication history for this paper can be accessed here:

http://www.biomedcentral.com/1471-230X/12/27/prepub
